# Long-term survival after solitary ocular metastasis following radical resection of esophagogastric junction carcinoma: a rare case report

**DOI:** 10.3389/fonc.2025.1650111

**Published:** 2025-11-20

**Authors:** Yangjun Gao, Yuru Zhai, Ning Ma, Xiaoling Zhang, Yunyi Du, Jun Zhao

**Affiliations:** 1 Department of Oncology, Changzhi People’s Hospital, The Affiliated Hospital of Changzhi Medical College, Changzhi, China; 2 Department of Ophthalmology, Changzhi People’s Hospital, The Affiliated Hospital of Changzhi Medical College, Changzhi, China

**Keywords:** esophagogastric junction carcinoma, ocular metastasis, oligometastasis, multimodal treatment, long survival

## Abstract

**Background:**

Isolated ocular metastasis from esophagogastric junction (EGJ) cancer is extremely rare, and no standardized therapeutic approach has been established to date. Reporting such cases may help clarify optimal management strategies for uncommon metastatic patterns.

**Case presentation:**

We describe a 44-year-old male patient who developed isolated ocular oligometastasis 10 months after radical EGJ cancer resection. Comprehensive evaluation confirmed left eye involvement. Following multidisciplinary team (MDT) discussion, a combined treatment plan was implemented, starting with systemic therapy consisting of docetaxel, tegafur/gimeracil/oteracil (S-1), and tislelizumab. After achieving disease stabilization, the patient underwent left eye enucleation with ocular prosthesis implantation, followed by adjuvant radiotherapy and maintenance S-1 chemotherapy. At a 55-month follow-up, he remains disease-free with an excellent performance status (PS 0).

**Conclusion:**

This case highlights that integrated multimodal therapy—including systemic and local interventions—can result in long-term survival for patients with isolated ocular metastasis from EGJ cancer. MDT-based, individualized treatment planning is essential for optimizing outcomes in rare metastatic scenarios and may inform future precision oncology approaches.

## Introduction

Gastric cancer ranks fifth worldwide in both incidence and mortality among malignant tumors (1). The esophagogastric junction (EGJ) is a frequent site of origin, and the tumor commonly spreads through direct invasion, lymphatic dissemination, or hematogenous metastasis. The peritoneum, distant lymph nodes, and liver are the most frequent metastatic sites (2), whereas ocular metastasis is exceedingly rare, with a reported incidence of less than 0.1% (3). Most lesions arise via hematogenous spread to the choroid, leading to visual impairment or blindness.

Because of its rarity, ocular metastasis from EGJ cancer lacks standardized diagnostic and therapeutic guidelines. Diagnosis relies on correlation with prior malignancy, imaging findings, and histopathology, and differentiation from primary ocular tumors such as uveal melanoma or inflammatory pseudotumor is essential. The prognosis is generally poor, and treatment is often palliative. Recent studies, however, suggest that multimodal management—including systemic therapy combined with local surgery or radiotherapy—may improve survival in patients with oligometastatic disease (4).

Here, we report a rare case of isolated ocularoligometastasis after radical surgery for EGJ cancer, successfully managed with immunochemotherapy and local therapy. This case highlights the potential benefit of combining systemic and local treatment for rare ocular metastases and provides insight into individualized strategies for EGJ cancer oligometastasis.

## Case report

A 44-year-old male patient presented in December 2019 with upper abdominal pain and dysphagia. Gastroscopy revealed carcinoma of the EGJ, and histopathology confirmed moderately differentiated adenocarcinoma. Serum CEA and CA72–4 levels were within normal ranges. The patient underwent combined thoracoscopic–laparoscopic radical resection, and postoperative staging was pT3N1M0, stage IIB, with proficient mismatch repair (pMMR) and negative HER-2 expression. From February to July 2020, he completed six cycles of SOX adjuvant chemotherapy [oxaliplatin 130 mg/m^2^ i.v. on day 1; tegafur/gimeracil/oteracil (S-1) 40 mg/m^2^ orally twice daily on days 1–14, every 3 weeks], with good tolerance and no complications.

**Figure 1:**
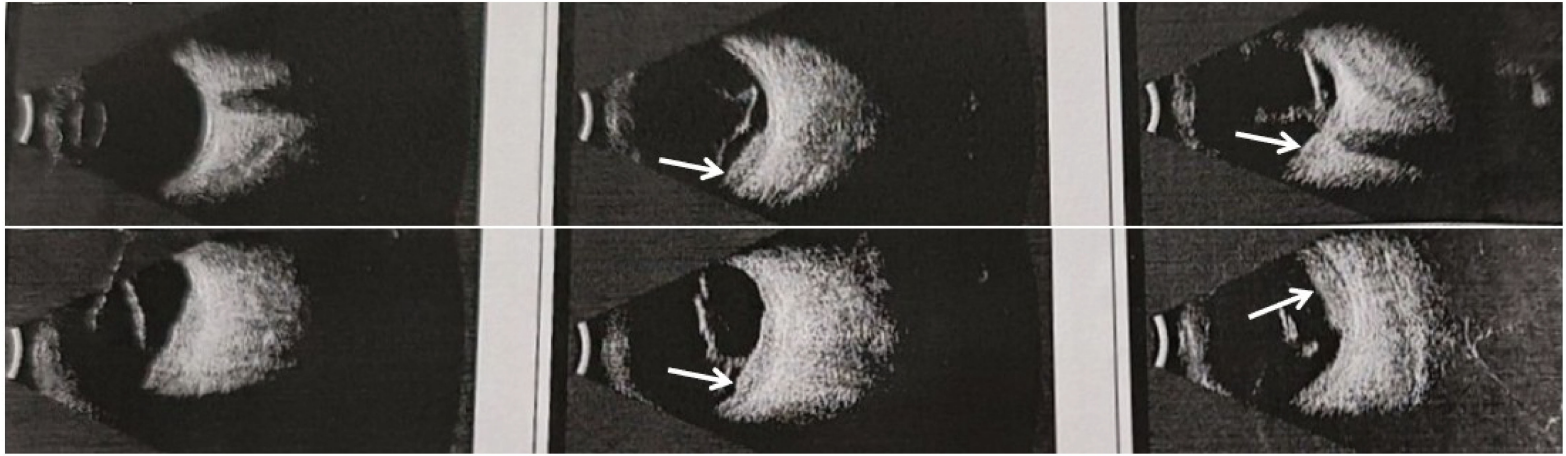
Ultrasonography of the left eyeball showing thickening of the choroid at the posterior pole, suggestive of an intraocular space-occupying lesion.

**Figure 2:**
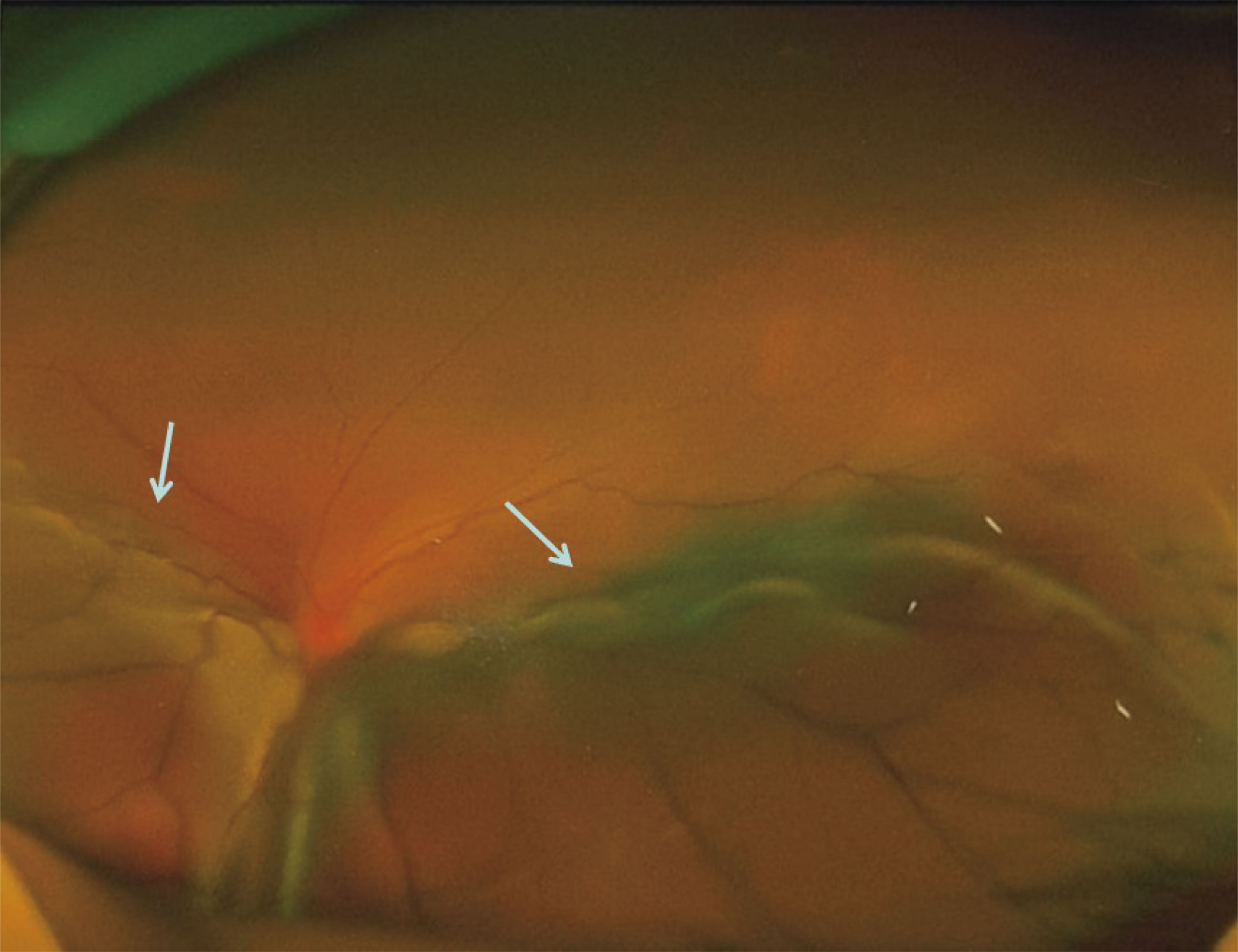
Fundus color photograph of the left eye showing elevation of the inferior retina involving the posterior pole.

**Figure 3:**
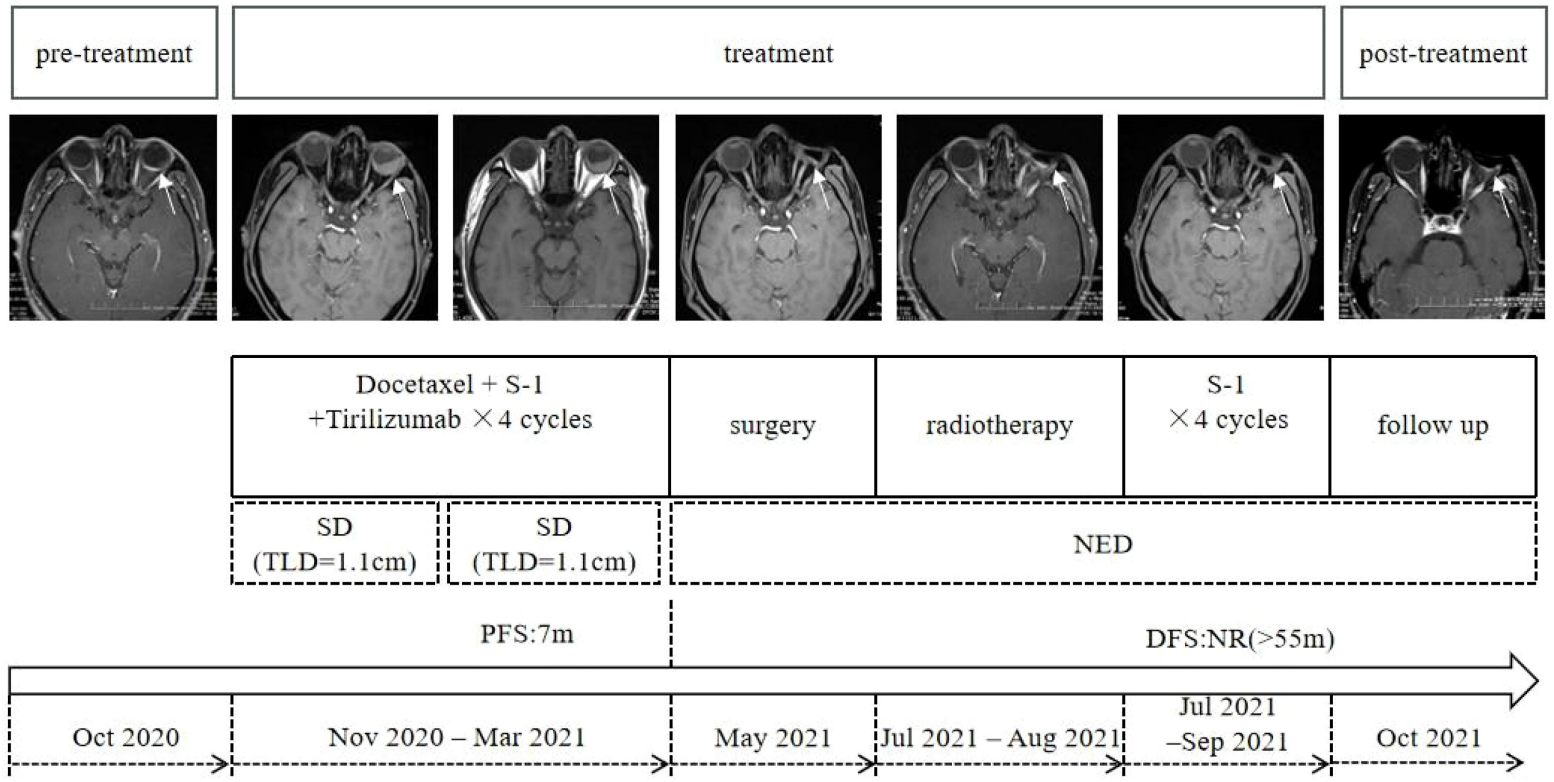
Timeline summarizing the patient’s diagnostic and therapeutic course for ocular metastasis (target lesion diameter, TLD).

In October 2020, the patient experienced progressive visual impairment in the left eye, leading to complete blindness within several weeks. Physical examination: blindness in the left eye. Ocular ultrasonography and fundus color Doppler imaging revealed a posterior intraocular mass and retinal detachment (**Figures 1**, **2**). Orbital magnetic resonance imaging (MRI) showed thickening of the posterior wall of the left eyeball (**Figure 3**), and PET-CT demonstrated increased FDG uptake confined to the ocular lesion without evidence of systemic metastasis. Serum CEA and CA72-4 remained within normal limits. Considering the patient’s medical history and distinctive imaging findings, a multidisciplinary team (MDT) consultation suggested the presence of distant metastasis. It was therefore recommended that systemic therapy be initiated first to achieve tumor control.

**Figure 4:**
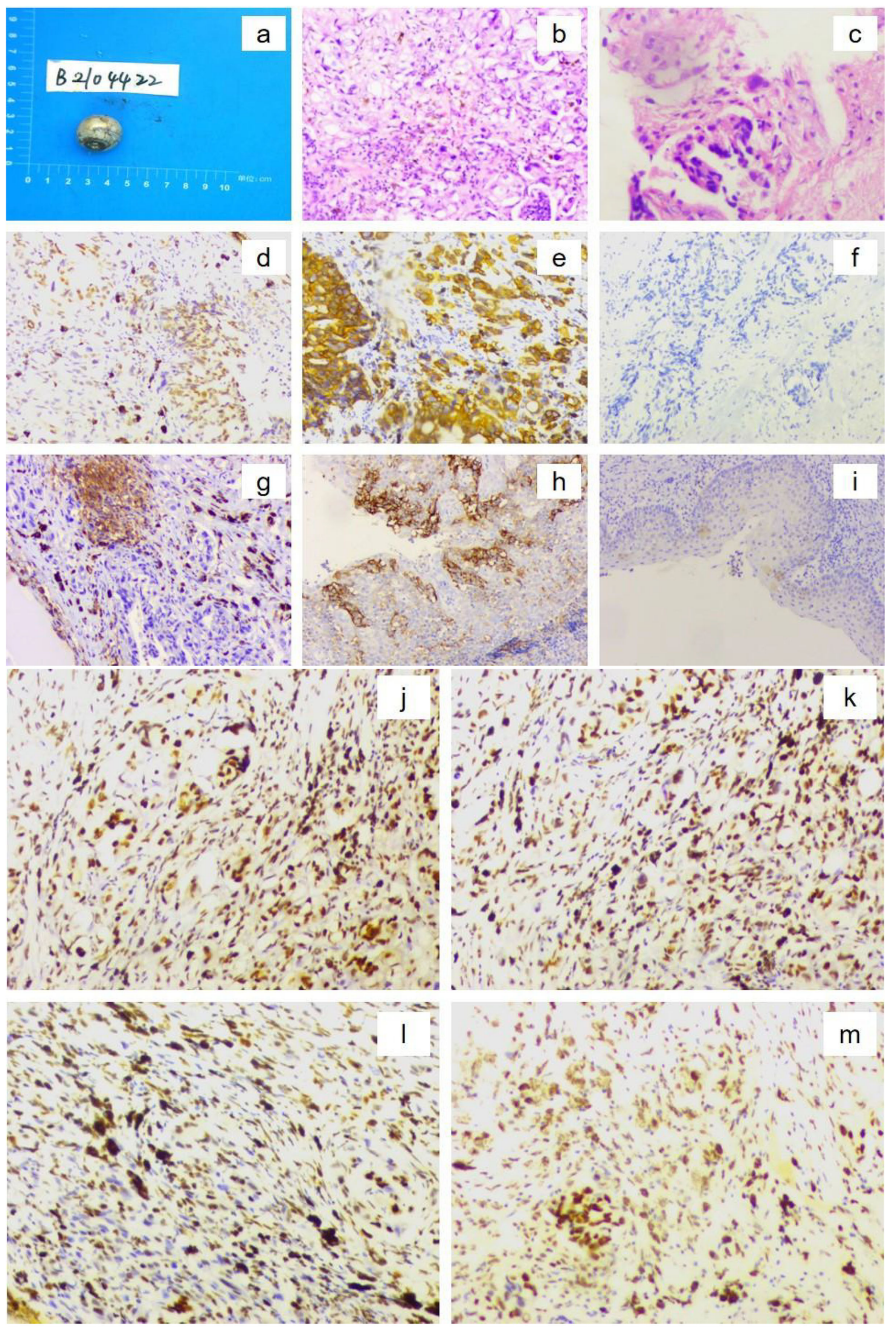
Postoperative histopathological findings of the left eyeball confirming metastatic gastric adenocarcinoma. [**(a)** Gross specimen of the resected eyeball; **(b)** Hematoxylin–eosin (H&E) staining showing numerous atypical cells (×10 objective); **(c)** H&E staining demonstrating tumor cell invasion into the optic nerve (×10 objective). Immunohistochemistry showing (×10 magnification): **(d)** CDX2 positive; **(e)** CK18 positive; **(f)** HER2 score 0; **(g)** PD-L1 expression, combined positive score (CPS) = 3; **(h)** PD-L1-positive control; **(i)** PD-L1-negative control; **(j)** MLH1 positive; **(k)** MSH2 positive; **(l)** MSH6 positive; and **(m)** PMS2 positive.].

From November 2020 to March 2021, the patient received combination systemic therapy consisting of docetaxel (75 mg/m^2^, day 1), S-1 (40 mg/m^2^, days 1–14), and tislelizumab (200 mg, day 1) every 3 weeks for four cycles. Follow-up orbital MRI was performed, and based on the immune-related Response Evaluation Criteria in Solid Tumors (irRECIST), the ocular lesion was assessed as stable disease (SD). The MDT concluded that the patient met the criteria for oligometastatic disease and that the tumor was under adequate systemic control; surgical resection was therefore recommended. In May 2021, the patient underwent left eye enucleation with orbital implant placement. Postoperative histopathology (**Figure 4**) confirmed metastatic gastric adenocarcinoma involving the eyeball, with a positive optic nerve margin. Immunohistochemistry showed PD-L1 combined positive score (CPS) = 3, HER2 negative, and proficient mismatch repair (pMMR). From 9 July to 17 August 2021, adjuvant local radiotherapy was administered to the orbital region (total dose 50 Gy in 25 fractions), followed by adjuvant chemotherapy with S-1 for 3 months. As of the end of April 2025, after 55 months of follow-up, the patient’s serum CEA and CA72-4 levels remain within normal ranges, and imaging studies show no evidence of recurrence or new metastases. His performance status (PS) score is 0, with good quality of life, no signs of anxiety or depression, and full ability to work. The patient was satisfied with the entire diagnostic and treatment process. The patient continues to be followed up regularly (treatment course shown in **Figure 3**).

## Discussion

Ocular metastasis from gastric cancer is extremely rare and generally associated with poor prognosis, often accompanied by multi-organ dissemination (3, 5). Its incidence is closely related to age, occurring predominantly in patients aged 80 years and older (6). The choroid, orbit, and extraocular muscles are among the most commonly affected sites (7–10). Because of the choroid’s rich vascular supply and slow blood flow, circulating tumor cells can easily lodge there and form metastatic deposits. Patients with ocular metastasis from gastric cancer often present initially with ocular symptoms (11, 12). Choroidal metastases typically manifest as decreased visual acuity or visual field defects (12, 13), while orbital metastases may present with proptosis, diplopia, or ptosis (7, 8). Diagnosis of ocular metastasis still relies on imaging modalities, such as MRI or CT, and definitive confirmation by histopathology (5, 6). Choroidal metastasis must be distinguished from primary choroidal melanoma and inflammatory lesions (12). Furthermore, many reported cases show concurrent distant metastases at the time of ocular involvement (3, 5), adding to the diagnostic complexity. In the present case, the patient developed a solitary ocular metastasis without evidence of other distant lesions, consistent with the biological characteristics of oligometastasis, thereby providing an opportunity for potentially curative multidisciplinary treatment.

For patients with gastric cancer, MDT discussion prior to treatment initiation is strongly recommended, as it allows for comprehensive assessment of the patient’s overall condition, better coordination of treatment sequence and modalities, and improvement in both quality of life and overall survival (14). According to the European OMEC-4 guidelines, for patients with metachronous oligometastatic gastric cancer and a disease-free interval (DFI) ≤ 2 years, systemic therapy should be administered first, followed by reassessment of local resectability (15). Systemic treatment options include surgery, radiotherapy, chemotherapy, targeted therapy, and immune checkpoint inhibitors (ICIs) (6, 16). Programmed death ligand-1 (PD-L1) combined positive score (CPS) is one of the key biomarkers for assessing potential benefit from ICI therapy; a higher CPS is generally associated with improved response and prognosis (17, 18). In the present case, the tumor exhibited PD-L1 expression with CPS > 1, suggesting a potential benefit from immunotherapy. Tislelizumab has demonstrated favorable efficacy both in the perioperative setting and as second-line therapy for gastric cancer (19, 20); therefore, a combination regimen of tislelizumab plus chemotherapy was selected for this patient. Following completion of two and four cycles of systemic therapy, timely MDT discussions were conducted, enabling appropriate evaluation of treatment response and facilitating surgical intervention at the optimal time.

Patients with ocular metastasis from gastric cancer generally have a poor prognosis and short median survival (3, 5). Studies have shown that once ocular metastasis occurs, the mean interval from diagnosis of the primary gastric tumor to the onset of ocular symptoms is approximately 25.4 months, while the average time from ocular symptom onset to death is only 3.3 months (21). A review of reports published over the past 5 years (**Table 1**) identified only three documented cases of ocular metastasis from gastric cancer, all accompanied by metastases to other organs. Treatment approaches in these cases included chemotherapy, surgery, and radiotherapy. Kato et al. reported a rare case of choroidal metastasis after surgery for EGJ carcinoma in an elderly male; despite active treatment, the survival time was only 16 months (22). Huang et al. described a middle-aged man with advanced gastric cancer and multiple metastases to the choroid and bone, who underwent systemic therapy followed by eye enucleation but died 2 months postoperatively (12). In contrast, the present patient developed solitary ocular oligometastasis. Initial chemo-immunotherapy effectively controlled disease progression and provided a therapeutic window for surgery. Subsequent postoperative radiotherapy eliminated residual tumor cells, and adjuvant chemotherapy suppressed potential micrometastases. This integrated multimodal approach ultimately led to long-term disease-free survival.

**Table 1:** Summary of reported cases of ocular metastasis published between 2020 and 2025.

No.	Reference	Age	Number of organ with metastases	Treatment	Survival
1	YanY H, et al, 2023 (12)	50	2	oxaliplatin and cetuximab, surgery	2 months after surgery
2	Rebollo, et al, 2023 (9)	82	2	hospice care	not mentioned
3	Kato C, et al, 2022 (22)	80	4	radiotherapy and oxaliplatin + S-1	16 months

This case provides several important insights: (1) Early recognition of intraocular symptoms and timely completion of comprehensive imaging studies can improve the diagnostic accuracy of ocular metastasis from gastric cancer. (2) For patients with oligometastatic disease, striving to achieve a no-evidence-of-disease (NED) status is essential; MDT deciding the treatment sequence is the key to improve the efficacy of treatment. (3) Although solitary ocular metastasis after EGJ carcinoma resection is extremely rare, a comprehensive treatment strategy combining systemic therapy with local surgery and radiotherapy can achieve long-term disease-free survival. This case highlights the crucial role of MDT collaboration in developing individualized therapeutic strategies for EGJ carcinoma and provides a valuable reference for managing similar cases. However, as a single case report, it has inherent limitations and lacks molecular-level mechanistic investigation. Further studies with larger cohorts are warranted to validate these findings.

## Data Availability

The original contributions presented in the study are included in the article/supplementary material. Further inquiries can be directed to the corresponding author.
